# Multipole resonance and Vernier effect in compact and flexible plasmonic structures

**DOI:** 10.1038/s41598-021-02333-9

**Published:** 2021-11-24

**Authors:** Yeonsoo Lim, Soo-Chan An, Hoon Yeub Jeong, Thi Hai-Yen Nguyen, Gangil Byun, Young Chul Jun

**Affiliations:** 1 Department of Materials Science and Engineering, Ulsan National Institute of Science and Technology (UNIST), Ulsan, 44919 Republic of Korea; 2 Department of Electrical Engineering, UNIST, Ulsan, 44919 Republic of Korea

**Keywords:** Nanophotonics and plasmonics, Metamaterials

## Abstract

Spoof surface plasmons in corrugated metal surfaces allow tight field confinement and guiding even at low frequencies and are promising for compact microwave photonic devices. Here, we use metal-ink printing on flexible substrates to construct compact spoof plasmon resonators. We clearly observe multipole resonances in the microwave frequencies and demonstrate that they are still maintained even under significant bending. Moreover, by combining two resonators of slightly different sizes, we demonstrate spectral filtering via the Vernier effect. We selectively address a target higher-order resonance while suppressing the other modes. Finally, we investigate the index-sensing capability of printed plasmonic resonators. In the Vernier structure, we can control the resonance amplitude and frequency by adjusting a resonance overlap between two coupled resonators. The transmission amplitude can be maximized at a target refractive index, and this can provide more functionalities and increased design flexibility. The metal-ink printing of microwave photonic structures can be applied to various flexible devices. Therefore, we expect that the compact, flexible plasmonic structures demonstrated in this study may be useful for highly functional elements that can enable tight field confinement and manipulation.

## Introduction

Surface plasmon polaritons (SPPs) in metal nanostructures can enable extreme confinement and guiding of light at optical frequencies^1–4^. However, at low frequencies, electromagnetic fields cannot penetrate into metals, and thus the plasmonic response and tight field confinement cannot be obtained. However, the introduction of subwavelength corrugations on a metal surface can allow a surface mode to be tightly bound to the metal surface even at microwave frequencies^5,6^. Electromagnetic fields in the corrugated region play a role similar to that of the field penetration of SPPs at optical frequencies.

Surface corrugation can be modeled as an effective medium with the Drude permittivity, where the plasma frequency is determined by the cutoff frequency of the groove waveguide^7^. The electromagnetic response of surface waves on corrugated metal surfaces can be controlled and designed by corrugation geometry. Such surface waves can be described by the dispersion relation similar to SPPs at optical frequencies. Therefore, we can use surface patterning to mimic SPPs even at low frequencies, called spoof (or designer) SPPs. In this way, the effective medium plasmonic metamaterials can be designed, and tight field confinement and manipulation can be still utilized in the low frequency region along with long propagation lengths of spoof SPPs. Moreover, such spoof SPP modes can exist in ultrathin films (down to almost zero thickness), called conformal SPPs^8,9^. Such conformal SPP modes can propagate through curved surfaces as well.

Spoof plasmon structures can also be applied to localized geometries, which are similar to localized surface plasmons (LSPs) at optical frequencies^10^. By circularly bending one-dimensional grooves, a low frequency analog of LSPs can be realized. Subwavelength electric and magnetic modes in spoof LSPs have been studied^11–21^, and the huge field enhancement in the gap between two plasmonic particles has been demonstrated too^20^. The strong field confinement and compact size of spoof LSPs make them ideal for electromagnetic sensing, similar to optical sensing with LSPs^9,22–25^. In addition, the resonance frequency and higher-order mode excitation can be readily controlled by adjusting structural parameters^26,27^.

Spoof surface plasmons can allow tight field confinement and guiding even in the low frequency region, such as the microwave region. On the other hand, there are strong and urgent needs for highly functional microwave photonic structures, such as antennas, filters, and sensors, in printed, low-cost flexible devices.

In the present study, we use metal-ink printing to construct compact spoof plasmonic resonators on flexible substrates. We clearly observe multipole resonances at microwave frequencies despite the limited conductivity of metal nanoparticle inks. In addition, by directly measuring the transmission in bent resonator structures, we also demonstrate that multipole resonances are maintained even for significant bending curvatures. This experimental observation is also confirmed using finite element simulations. Moreover, we realize more functional elements using the Vernier effect in two coupled plasmonic resonators. By combining two resonators of slightly different sizes, we demonstrate spectral filtering via the Vernier effect. In particular, with adjusted probe locations, we significantly increase the transmission contrast between the target resonance and the other resonances. Therefore, we selectively address a target higher-order resonance in the Vernier structure while suppressing the other resonances. We also investigate the index-sensing capability of a higher-order resonance in printed plasmonic resonators. Higher-order resonances are spectrally sharper than a dipole resonance, and thus they are favored for various microwave components and sensing. In a single resonator, a gradual change in the resonance frequency occurs when the index of the surrounding medium changes. In the Vernier structure, we also control the resonance amplitude by controlling a resonance overlap between two coupled resonators. The transmission amplitude can be maximized at our target index, and this can provide more functionalities and increased design flexibility.

**Figure 1:**
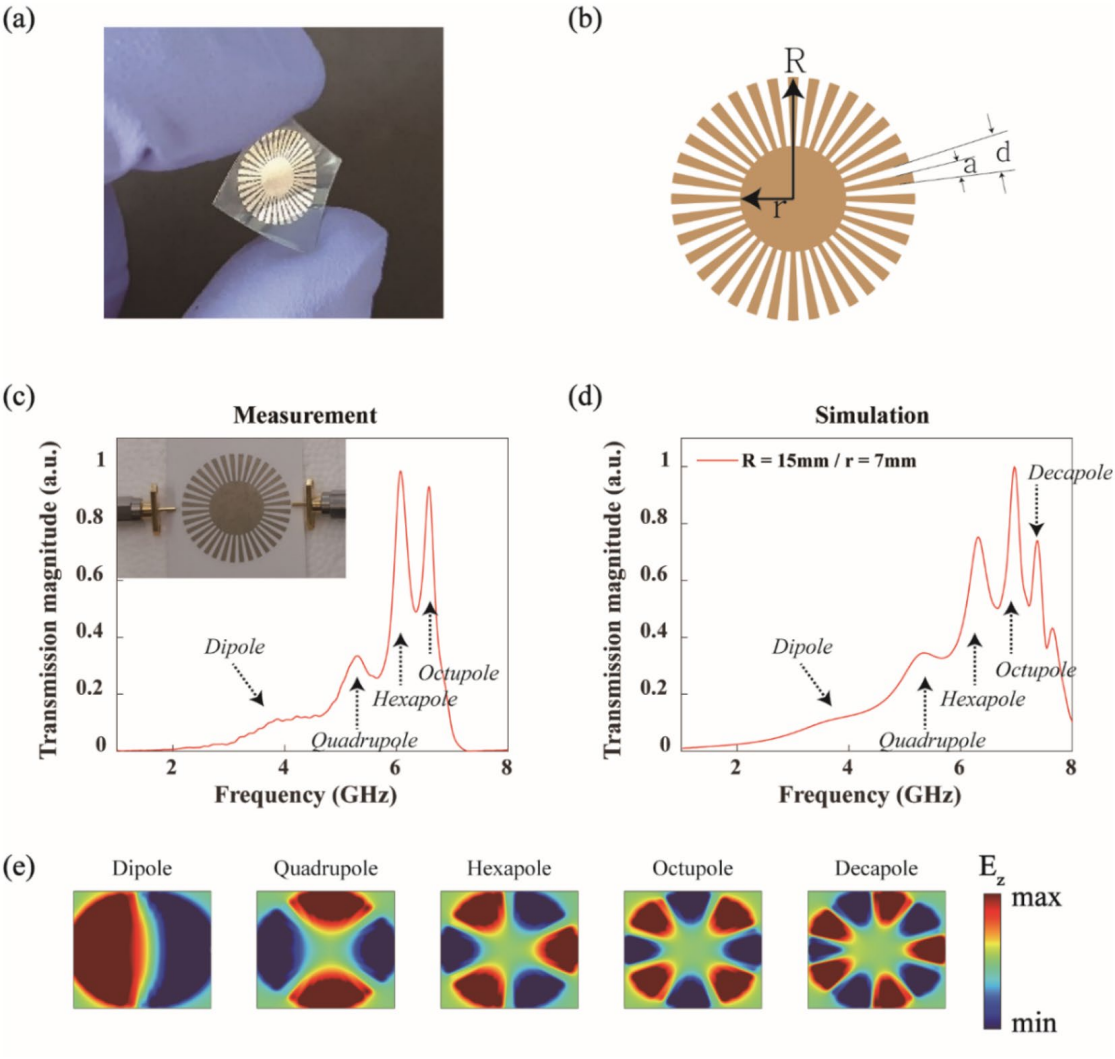
Metal ink printing of spoof plasmonic resonators on flexible substrates. (**a**) Picture of a plasmonic resonator on a flexible substrate. (**b**) Design parameters of a spoof plasmon resonator: the outer radius *R,* the inner radius *r*, the groove period *d*, the groove width *a* (= *d*/2), the number of grooves *N* (= 2π*R/d*). (**c**) and (**d**) Measured and simulated transmission spectra for a plasmonic resonator printed on paper (*R* = 15 mm, *r* = 7 mm, and *N* = 36). The black arrows indicate the position of multipole resonances. In simulations, the substrate was intentionally omitted to simplify the mesh condition. (**e**) Field profiles (E_z_) obtained from simulations. Measured 1 mm above the sample surface.

Figure 1a shows one example of plasmonic resonators printed on a flexible substrate. The metal-ink printing of microwave photonic structures can be widely usable in various flexible devices^28^. Therefore, we expect that the compact, flexible plasmonic resonators we demonstrate here could be useful for highly functional elements, such as antennas, filters, and sensors in printed, inexpensive flexible devices.

## Results and discussion

Compact plasmonic resonators were printed using silver nanoparticle inks. Metal nanoparticle inks can be used in inkjet printers and facilitate the fabrication of microwave components on various substrates^29–31^. For our experiments, we selected photopaper as a substrate because of its porosity, which helps the ink soak into the paper via capillaries. After printing, the metal-ink sample was annealed on a hot plate to increase the conductivity of silver-ink patterns. Figure S1 in “Supplementary Information” shows the scanning electron microscope (SEM) images of printed patterns under different annealing conditions. With increased temperatures, silver nanoparticles grow larger and agglomerate. Residual organic materials between silver nanoparticles are also removed during this annealing. Our samples were annealed at 180 °C for 3 min. By further increasing the annealing temperature and time, the conductivity was increased further. However, we found that the photopaper can be damaged (see Fig. S1). Therefore, we used this annealing condition for the sample fabrication. See “Methods” for more details regarding the sample fabrication.

Figure 1b shows the plasmonic resonator design we used in this study, where *r* is the radius of the inner circle fully filled with metal and *R* is the total radius including the outer grooves. The groove width *a* is set as half of the groove period *d*. The required resolution for this plasmonic structure can be readily achieved with conventional inkjet printers. Although the conductivity of the printed sample is smaller than that of copper or aluminum lines (> 10^7^ Ω^−1^ m^−1^) on conventional printed circuit boards (PCBs), printed metal-ink patterns still show clear multipole resonances. In our measurements, two probes were facing each other at the opposite side of the plasmonic resonator, and the transmission amplitude (|S_21_|) was measured using a vector network analyzer (VNA). Figure 1c shows the measured transmission spectrum for the plasmonic pattern with *R* = 15 mm and *r* = 7 mm. The inset in Fig. 1c shows the picture of our measurement configuration. Multiple, clearly separated resonances are observable. By comparing to a simulated spectrum, the measured resonance peaks are identified as dipole, quadrupole, hexapole, and octupole resonances. By curve fitting, the Q factors of hexapole and octupole resonances are determined to be 18 and 31, respectively.

The measured spectrum is very similar to the simulated one shown in Fig. 1d. We used the same probe configuration in our numerical simulation. Simulations were performed without a substrate (paper) to simplify the mesh condition. Except for a small frequency shift between the measurement and simulation, they are in good agreement. In the simulated spectrum, we also note the decapole resonance, which is not clearly visible in our measurement. Figure 1e shows the field profiles for multipole resonances (electric dipole, quadrupole, hexapole, octupole, and decapole) obtained from the simulation. The number of nodes (i.e., field zero lines) gradually increases for higher-order resonances.

These multipole resonances occur by a spoof SPP wave traveling along the circular shape of the compact plasmonic structure. Therefore, multipole resonances are excited as a form of standing waves^10,11,13^, when SPP waves approximately satisfy

1 $$n{\lambda }_{sp}\approx 2\pi R,$$

where positive integer *n* is the azimuthal mode number, *λ*_*sp*_ is the wavelength of spoof SPPs, and *2πR* is the circumference of the structure. It is also known that spoof LSP structures can also support radial higher-order modes for a very small core radius^26^. However, in our current work, we only use the fundamental modes in the radial direction.

Multipole resonances in a spoof LSP resonator can be tailored using asymptote resonance tuning. Figure S3 in “Supplementary Information” shows more details for spoof LSP design; it shows how the dispersion curve moves with the inner radius r. Figure S3a,b show the design and probe configuration. The dispersion relation of a two-dimensional (2D) spoof LSP structure (i.e., an infinitely long cylinder) can be obtained from an analytic relation. Using Mie scattering and an effective medium approximation, the dispersion relation of spoof LSPs in such a 2D structure can be approximately written as^7,26^,

2 $$\frac{n}{R}={k}_{0}\sqrt{1+{\left(\frac{a}{d}\right)}^{2}{\mathit{tan}}^{2}\left({k}_{0}h\right)},$$

where *k*_*0*_ is the wavevector in free space and *h* = *R* − *r*, assuming the background medium is air. In Eq. (1), *n/R* = *2π/λ*_*sp*_ = *k*_*sp*_. Thus, the right side of Eq. (2) corresponds to the wavevector *k*_*sp*_ of the propagating SPPs. This can be understood from the fact that a spoof LSP resonator is constructed by circularly bending a SPP waveguide^26^. Finally, the asymptote frequency of spoof LSPs is obtained as^7,10,12^

3 $${\omega }_{a}=\frac{\pi c}{2h{n}_{g}},$$

where *n*_*g*_ is the refractive index filling the groove. Using Eq. (2), we calculated the dispersion relation of spoof LSPs, as shown in Fig. S3c. The outer radius is fixed as *R* = 15 mm, whereas the inner radius *r* is varied from 3 to 12 mm with an increment of Δ*r* = 1 mm. Increasing *r* (or decreasing *h*) results in a higher asymptote frequency (ω_a_) and the blueshift of resonances. Furthermore, the slope of the dispersion relation gradually decreases as *r* increases. As a result, the decreasing *h* leads to not only resonance shifts, but also the appearance of higher-order azimuthal modes^32^. For ultrathin films, the dispersion curves deviate from this ideal 2D structure, but the main features remain similar. Figure S3d shows the simulated transmission spectra for an ultrathin structure with varying inner radii. As the inner radius increases, we can see that the multipole resonances blueshift as expected from the dispersion relation. The number of observable multipole resonances is also varied. Figure S3e shows field profiles for several higher-order modes, which are indicated by arrows in Fig. S3d.

Metal-ink printing on flexible substrates is suitable for microwave component fabrication in various flexible devices. In fact, conformal SPP waveguides have been studied, and it has been demonstrated that spoof SPPs can propagate along curved surfaces with very low propagation losses^8,9^. Here, we consider the direct bending of a compact plasmonic resonator and, through both experiment and numerical modeling, demonstrate that multipole resonances are still maintained despite significant bending.

**Figure 2:**
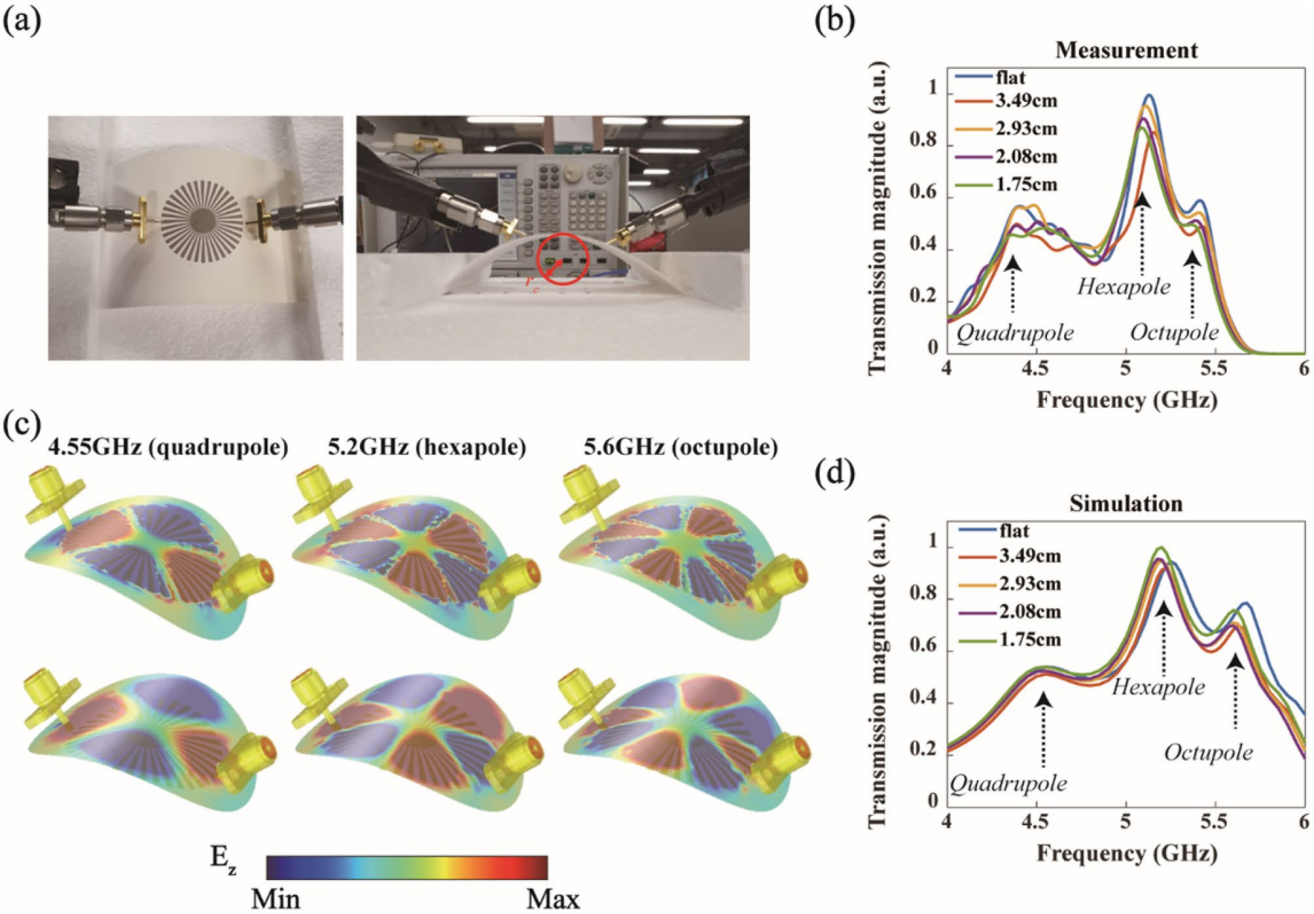
Direct bending of a spoof plasmonic resonator (*R* = 15 mm, *r* = 4.5 mm)*.* (**a**) Picture of experimental configuration (Left: top view, Right: side view. The definition of the radius of curvature *r*_*c*_ is also defined). (**b**) Measured transmission spectra for different bending curvatures (radius of curvature *r*_*c*_). The black arrows indicate the position of multipole resonances. (**c**) Simulated mode profiles (E_z_) for quadrupole, hexapole, and octupole resonances in a bent resonator (*r*_c_ = 2.08 cm). The upper panel is the field profile measured 0.5 mm above the structure, while the lower panel is measured 3 mm above the structure. (**d**) Simulated transmission spectra.

A compact spoof LSP resonator of *R* = 15 mm and *r* = 4.5 mm was printed on paper. As our resonator is printed on paper, it can be easily bent, as shown in Fig. 2a. The printed resonator was placed on top of a Styrofoam plate (the refractive index of which is close to 1 at microwave frequencies) and bent by two side Styrofoam blocks. Figure 2a shows the top and side views of the experiment configuration. By adjusting the distance between two Styrofoam blocks, we varied the curvature of the bent resonator. Figure 2b shows the measured transmission amplitude spectrum for several different curvatures. We observe that even when the radius of curvature *r*_*c*_ decreases significantly from infinity (flat) to *r*_*c*_ = 1.75 cm, the measured multipole resonances are still maintained.

We also confirmed this bending behavior using finite element simulations. Details regarding simulation conditions can be found in the “Methods” section. Figure 2c shows the simulated mode profiles for quadrupole, hexapole, and octupole resonances in a bent resonator at their corresponding resonance frequencies (4.55, 5.2, and 5.6 GHz, respectively). The upper panel is the field profile measured 0.5 mm above the structure, whereas the lower panel is measured 3 mm above the structure. These field profiles (E_z_) clearly confirm that multipole resonances can be maintained in a curved spoof plasmon resonator. Figure 2d shows the simulated transmission spectrum for several bending curvatures. Except for a slight frequency shift and amplitude modulation, the quadrupole, hexapole, and octupole resonances are maintained for all bending curvatures considered. The simulation results agree reasonably well with experimental ones in Fig. 2b.

**Figure 3:**
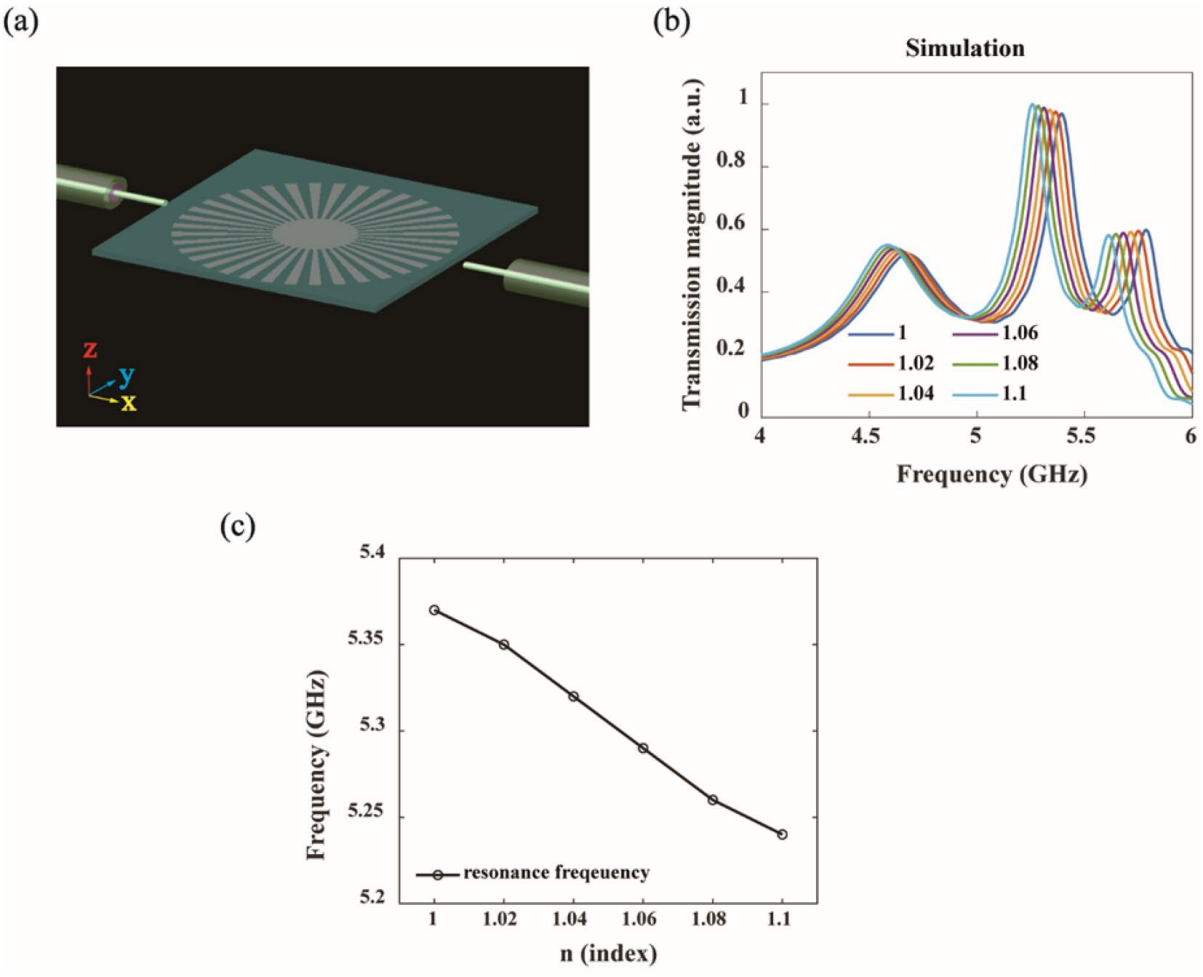
Sensing capability of printed plasmonic resonators (simulations). (**a**) Simulation configuration: a plasmon resonator (R = 15 mm, r = 4.5 mm) is covered by a thin dielectric layer (thickness = 1 mm) of varying indices. (**b**) Simulated transmission spectra for indices of 1, 1.02, 1.04, 1.06, 1.08, and 1.1. (**c**) Resonance frequency of the hexapole mode as a function of the refractive index of the thin dielectric layer.

Spoof plasmon structures can confine electromagnetic fields tightly even at microwave frequencies and thus they can be useful for electromagnetic sensing. To test the sensing capability of plasmonic resonators, we have conducted more simulations; a spoof plasmon resonator is covered by a thin dielectric layer (thickness = 1 mm) of varying indices (1, 1.02, 1.04, 1.06, 1.08, and 1.1). Figure 3a shows our simulation configuration and Fig. 3b is the simulated transmission spectrum for different indices. As the index increases, the resonance peak redshifts and the amplitude changes monotonically, as commonly observed in index-sensing experiments^22,25,33–35^. Figure 3c shows how the resonance frequency of the hexapole mode changes with the index variation, and we obtain the sensitivity of 1.3 GHz/RIU from the slope.

Thus far, we have considered multipole resonances in single plasmonic resonators. More functional elements can be constructed using coupled resonators. For example, we can filter out unwanted resonance peaks and isolate a target resonance using the Vernier effect in coupled plasmonic resonators. The metal-ink conductivity is typically lower than that of metal lines on PCBs, and thus resonances in printed samples are often broadened. The peaks of multiple resonances may partially overlap and may not become so clear. In our case, we can isolate a single higher-order resonance peak via the Vernier effect, which could be useful for many metal-ink printed devices.

The Vernier effect in transmission occurs because of the energy transport via spectrally overlapped resonances^36–39^. When two resonators of slightly different sizes are coupled via a nearfield overlap, only the overlapped resonance peaks have a prominent transmission amplitude, whereas the other spectrally misaligned resonances are suppressed. There have also been optical sensing studies based on the Vernier effect in the optical frequency range^36,38,39^.

**Figure 4:**
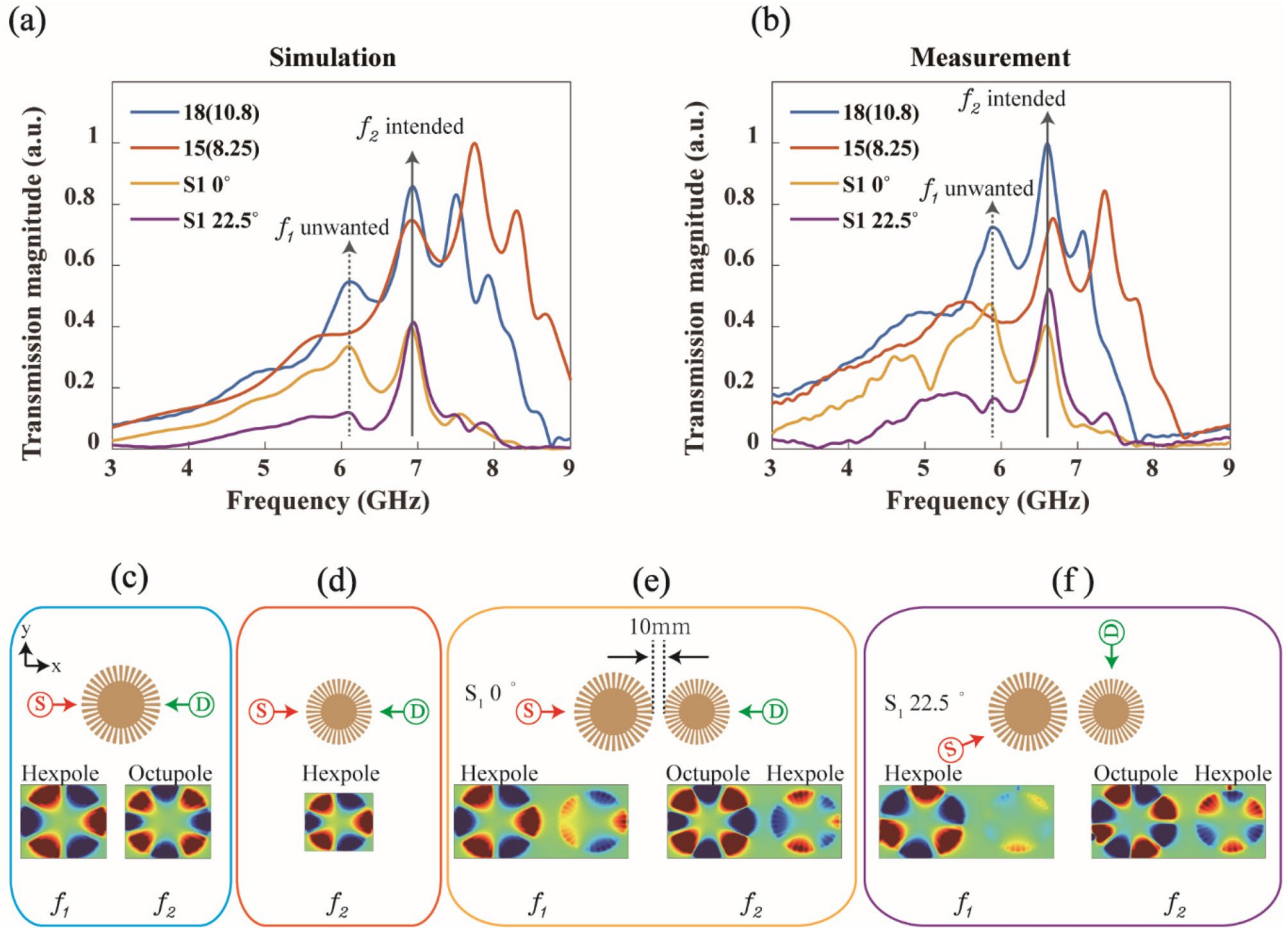
Vernier effect in a coupled plasmonic resonator which consists of larger (*R* = 18 mm, *r* = 10.8 mm) and smaller (*R* = 15 mm*, r* = 8.25 mm) resonators. (**a**) and (**b**) are the simulated and measured transmission spectra. The blue and orange curves are the spectra from individual larger and smaller resonators, respectively. (**c**) and (**d**) show the probe configuration and simulated field profiles corresponding to blue and orange curves. The yellow and purple curves are the transmission spectra from the Vernier structure with the probe configurations shown in (**e**) and (**f**), respectively. The solid black arrow (*f*_*2*_ intended) is the target resonance position in the current Vernier structure. With adjusted probe locations, the transmission contrast between the target resonance (*f*_*2*_ intended) and the other resonances is significantly increased. The field profiles in (**c**)–(**f**) are obtained at frequencies *f*_1_ and *f*_2_.

Here, we demonstrate the Vernier effect in coupled plasmonic resonators of two different sizes; for a larger resonator, the outer and inner radii are *R*_*1*_ = 18 mm and *r*_*1*_ = 10.8 mm, respectively, while *R*_*2*_ = 15 mm, *r*_*2*_ = 8.25 mm for a smaller one. We chose this dimension so that the multipole resonances of the smaller resonator slightly shift to higher frequencies, causing a higher-order mode of the larger resonator to spectrally overlap with a lower-order mode of the smaller resonator. Figure 4a,b show the simulated and measured transmission spectra, respectively. To measure the transmission amplitude, we first set the two probes facing each other [see the configuration in Fig. 4c,d]. The blue and orange curves in Fig. 4a are the simulated spectra from the individual larger and smaller resonators, respectively. Each resonator shows clear multipole resonances, and it is designed that the octupole of the larger resonator spectrally matches the hexapole of the smaller resonator (referred to as ‘*f*_*2*_ intended’ in the figure).

The transmission in the coupled Vernier structure is shown as a yellow curve [see also the configuration in Fig. 4e]. At the target frequency (‘*f*_*2*_ intended’), the transmission peak is clearly visible as expected. In the higher frequency region, the higher-order modes are clearly suppressed. However, we notice that, in the lower frequency region, the spectral filtering is not perfect, and still an unwanted transmission peak remains (for example, see the peak indicated as ‘*f*_*1*_ unwanted’ on the yellow curve). Because of broader resonances in lower-order modes (quadrupole in the smaller resonator), the unwanted transmission peak still remains. However, we find that, if we rotate the source and detector probes as shown in Fig. 4f, we can suppress this low frequency peak as well. The simulated transmission spectrum for this probe configuration is also shown in Fig. 4a (purple curve). In this case, the source probe is rotated by 22.5°, while the detector probe is rotated by 90°. Both are rotated in the counter-clockwise direction [see the probe configuration in Fig. 4f]. Now, only the target resonance of approximately 7 GHz remains strong. Except for one target resonance at *f*_*2*_, all other peaks are significantly suppressed. Therefore, we can selectively address a higher-order resonance in the plasmonic Vernier structure. We also verified this behavior experimentally, as shown in Fig. 4b. Because of the absence of a substrate in the simulations, the resonance peaks in measurements are slightly redshifted compared with those in the simulations. With the exception of this spectral shift, experiments agree well to simulations overall. With the rotated probe configuration (purple curve), a single resonance peak is dominant and others are strongly suppressed.

We can understand this behavior from the field profiles in Fig. 4e,f. When source and detector probes face each other (Fig. 4e), hexapole and octupole mode energies in the larger resonator can be transferred to the smaller resonator at both frequencies *f*_*1*_ and *f*_*2*_. See the field profiles in Fig. 4e. Therefore, we still have unwanted low frequency energy. However, with the rotated source probe (Fig. 4f), the mode profile in the larger resonator is also rotated accordingly (see also Fig. S5 in “Supplementary Information”). Note that the source probe is a driving point of the mode and becomes an antinode (i.e., field maximum) position of the mode. Then, the mode of the smaller resonator is also rotated accordingly, because it is excited by the larger resonator. We also note that the nearfield coupling between two resonances changes from pole-pole to node–node coupling^19^; compare the field profiles at *f*_*2*_ in Fig. 4e,f. Now, only at the target frequency *f*_*2*_, mode energy can be efficiently transferred to the new detector probe position [see field profiles in Fig. 4f]. Therefore, we can isolate the single target resonance at *f*_*2*_ [purple curve in Fig. 4a,b].

In our Vernier structure, the separation between the two resonators is fixed at 10 mm so that they remain in the weak coupling regime. If the separation decreases down to the order of ~ 1 mm, strong coupling occurs between two resonators, and each resonance peak in the transmission spectrum splits into two hybrid modes (bonding and anti-bonding modes)^17,18,20,21^. This complicates transmission behavior and can worsen spectral filtering at a single target frequency. Thus, to achieve clear spectral filtering in our experiment, we ensured that there was a sufficiently large gap between two resonators. This gap size results in the reduction of transmission amplitude. However, as shown in our measurement results [yellow and purple curves in Fig. 4b], the transmission amplitude remains strong enough to obtain the spectral filtering.

**Figure 5:**
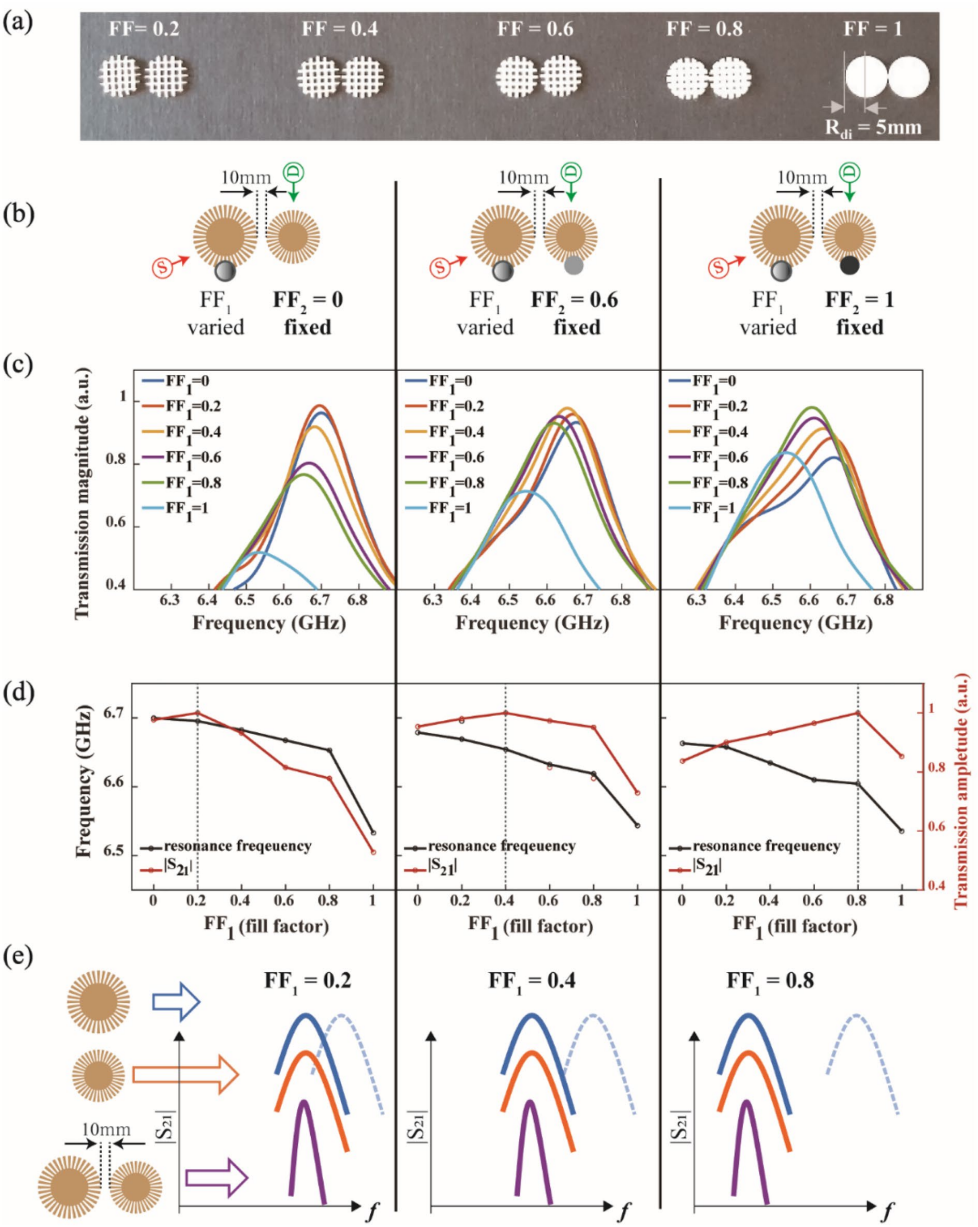
Index sensing test in the Vernier structure. All results are experimental data. A slight mismatch between resonances in two single resonators is initially introduced to induce the amplitude tuning of the filtered peak (See Fig. S4 in “Supplementary Information”). (**a**) Picture of sensing elements with the different fill factors (*FF*) of 0.2, 0.4, 0.6, 0.8, and 1, respectively, which approximately correspond to the refractive indices of 1.14, 1.28, 1.42, 1.56, and 1.7. (**b**) shows experimental configurations: a sensing element (fill factor *FF*_*1*_) is introduced on the left resonator and gradually varied, while fixing an element on the right resonator (fill factor *FF*_*2*_ fixed). The left, middle and right columns correspond to the case where *FF*_*2*_ is fixed as 0, 0.6, and 1, respectively. (**c**) Measured transmission spectra. (**d**) Resonance frequency and amplitude change. The amplitude becomes maximum at different *FF*_*1*_ values for each case (0.2, 0.4, and 0.8 for the left, middle, and right columns, respectively). (**e**) Schematic illustration of index sensing in the Vernier structure. The blue, orange, and purple lines correspond to the larger, smaller, and coupled resonators, respectively. With varying *FF*_*1*_, the blue line gradually redshifts (from dashed to solid ones), while the orange line remains fixed at a frequency value (determined by *FF*_*2*_). In each case, the transmission maximum of the Vernier structure is achieved at different *FF*_*1*_.

Lastly, we checked the sensing capability of a coupled Vernier structure as follows. Dielectric disks (radius 5 mm and height 1 mm) were prepared using three-dimensional (3D) printing. The fill factor (*FF*) in dielectric disks was varied during 3D printing so that the refractive index varies gradually [see Fig. 5a]. The printed sensing elements are much smaller than the measurement wavelength (λ = 60 mm at 5 GHz). The refractive index of the 3D printing material (VeroWhite) was measured to be ~ 1.7. Thus, the refractive index *n* of our dielectric disks can be estimated by the effective medium approximation: $n\approx 1\times \left(1-FF\right)+1.7\times FF$. By increasing *FF* from 0 to 1, we achieved a very wide range of refractive index values from 1 to 1.7, and we could experimentally investigate the response of plasmonic resonators in the wide range of index values. Figure 5a shows the printed dielectric disks when *FF* = 0.2, 0.4, 0.6, 0.8, and 1, which approximately correspond to the refractive indices of 1.14, 1.28, 1.42, 1.56, and 1.7, respectively. This time, we slightly adjusted the radius of the larger resonator (*R*_1_ = 17.8 mm, *r*_1_ = 10.68 mm) to induce a slight mismatch of the resonance frequency between two single resonators at the target frequency *f*_*2*_. This mismatch enables the amplitude tuning of the filtered peak when the index changes.

We conducted experiments by varying sensing elements on the larger (left) resonator (i.e., *FF*_*1*_ varied) while fixing elements on the smaller (right) resonator (i.e., *FF*_*2*_ fixed). Figure 5b explains the experimental configuration; the left, middle and right columns correspond to the case where *FF*_*2*_ is fixed as 0, 0.6, and 1, respectively. The measured transmission spectrum is shown in Fig. 5c. Figure 5d shows how the resonance frequency and amplitude change in each case with *FF*_*1*_. The frequency of the filtered resonance peak gradually redshifts. However, the amplitude reaches its maximum at different *FF*_*1*_ values for each case (0.2, 0.4, and 0.8 for the left, middle, and right columns, respectively). Figure 5e schematically explains the behavior; the blue and orange lines correspond to the transmission amplitude of larger and smaller resonators, respectively, and the purple line corresponds to the coupled Vernier structure. With varying *FF*_*1*_, the blue line gradually redshifts (from dashed to solid lines), whereas the orange line (the resonance of the smaller resonator) remains fixed at a frequency value that is determined by *FF*_*2*_. Therefore, in each case, we achieved an overlapped resonance at different *FF*_*1*_. This results in the amplitude tuning of index sensing.

In a Vernier structure consisting of two coupled resonators, the transmission amplitude of a filtered resonance peak reaches its maximum when two resonances spectrally overlap. The overlapped peak will respond sensitively to the resonance shift of each single resonator. Therefore, depending on the spectral shift of each resonance peak, the transmission amplitude can either increase or decrease. Thus, the transmission amplitude can also be engineered; e.g., it can be maximized at our target condition. Therefore, more functionalities and increased design flexibility can be obtained.

The metal-ink printing of spoof plasmonic structures has unique merits for microwave photonic components. Because the geometrical parameters of plasmonic structures are millimeters in size in a few gigahertz frequency range, conventional inkjet printers are sufficient to provide enough structural accuracy. By employing more advanced printing technology, the operating frequency can be increased further^40^. Strong field confinement in spoof plasmonic structures make them ideal for compact functional elements and electromagnetic sensing. Moreover, printing on paper is advantageous for low-cost, disposable sensing devices.

The future wireless communication requires higher frequency bands to further increase data capacity^41,42^, and highly functional microwave components are expected to be deployed in numerous wireless devices^43,44^. Spoof plasmonic structures can enable tight field confinement and manipulation from the microwave to the terahertz region. Therefore, compact spoof plasmonic structures on flexible substrates may play an important role in the functional components of various flexible devices.

## Conclusions

In this work, we used metal-ink printing on paper to create compact, flexible spoof plasmonic resonators. We clearly observed multipole resonances in the microwave frequencies and demonstrated that multipole resonances are still maintained even for significant bending curvatures. Moreover, we realized more functional elements using the Vernier effect in two coupled plasmonic resonators. By combining two resonators of slightly different sizes, we demonstrated spectral filtering via the Vernier effect. With adjusted probe locations, we could significantly increase the transmission contrast between the target resonance and the other resonances. In this way, we were able to selectively address a target higher-order resonance while suppressing the other modes. We also investigated the index-sensing capability of printed plasmonic resonators. In the Vernier structure, we were able to control the resonance amplitude as well by controlling a resonance overlap between two coupled resonators. This can provide more functionalities and increased design flexibility.

The metal-ink printing of microwave photonic structures can be applied to various flexible devices. However, the metal-ink conductivity is typically lower than that of conventional metal lines on PCBs. Thus, resonance peaks in metal-ink-printed structures get broader, and it may become more difficult to individually address higher-order modes. In our work, we demonstrated the isolation of a single resonance peak via the Vernier effect, and this can be useful for microwave components in many metal-ink printed devices. Tight field confinement and manipulation in compact, flexible plasmonic structures may be important for many functional elements in flexible devices, such as antennas, filters, and sensors.

## Methods

### Metal-ink printing on paper

Spoof plasmonic resonators were fabricated using an inkjet printer (Epson, Stylus C88+) and silver nanoparticle inks (Novacentrix, JS-B25P). Silver nanoparticles were printed on Kodak photo paper (thickness ~ 230 μm). After printing, thermal sintering was conducted in a heat oven at 180 ℃ for 3 min (see Fig. S1 in the “Supplementary Information”). The height of the printed patterns was determined to be ~ 1 μm using a surface profiler (KLA Tencor, P6). The conductivity of annealed samples was measured to be about 2.5 × 10^6^–4.9 × 10^6^ Ω^−1^ m^−1^, depending on the annealing and metal-ink conditions. Resonances in the high frequency range are more likely to be affected by the printing resolution, because high-frequency (higher-order) resonances get shorter in wavelength and feel more fine features.

### Microwave measurement

A coaxial probe, which consists of a coaxial cable and a SMA connector, was used for transmission measurements. One end of the coaxial cable was connected to a VNA, while the other was connected to the SMA connector for impedance matching. Two probes were deployed at opposite sides of the sample to measure the transmission amplitude (|S_21_|) (see also Supplementary Fig. S2b and the description therein).

### Bending simulation

Fininte element analysis (COMSOL multiphysics) was used to simulate the bending of a spoof plasmonic resonator (R: 15 mm, r: 4.5 mm) printed on flat paper (9 cm × 6 cm, thickness: 230 μm). Paper was pushed at both ends with the radius of the bending curvature (*r*_*c*_) of 3.49, 2.93, 2.08 and 1.75 cm. After taking a picture of the bent sample, the curvature was determined from the image using MATLAB codes ^45^. Then, the radius of curvature *r*_*c*_ was obtained from ${r}_{c}=\frac{1}{curvature}$ at the center of the sample (see Fig. 2a). A bent shape was constrained by a wall (5 mm × 600 mm × 20 mm) on both sides, which was a rigid domain (i.e., there was no shape transformation during the bending of the resonator). Then, the curved geometry from the mechanical simulation module was imported to the electromagnetic module for transmission simulations. The source and detector ports were attached to a curved spoof plasmon structure. The outer sphere was set as the scattering boundary condition. Electromagnetic simulations showed clear multipole resonances as shown in Fig. 2c,d.

## Supplementary Information

Supplementary Information.

## Data Availability

The raw data and the processed data required to reproduce the findings in the current study are available from the corresponding author on reasonable request.
