# Supplementary material for: Multipole resonance and Vernier effect in compact and flexible plasmonic structures
Source: Sci Rep. 2021 Nov 24;11:22817. doi: 10.1038/s41598-021-02333-9 (PMC8613286; doi:10.1038/s41598-021-02333-9)
Supplement: Supplementary file 1 — Supplementary Information. [file 41598_2021_2333_MOESM1_ESM.docx]

**Supplementary Information**

**Multipole resonance and Vernier effect in compact, flexible plasmonic structures**

Yeonsoo Lim^1^, Soo-Chan An^1^, Hoon Yeub Jeong^1^, Thi Hai-Yen Nguyen^2^, Gangil Byun^2^, and Young Chul Jun^1^

^1^Department of Materials Science and Engineering, Ulsan National Institute of Science and Technology (UNIST), Ulsan 44919, Republic of Korea

^2^Department of Electrical Engineering, UNIST, Ulsan 44919, Republic of Korea


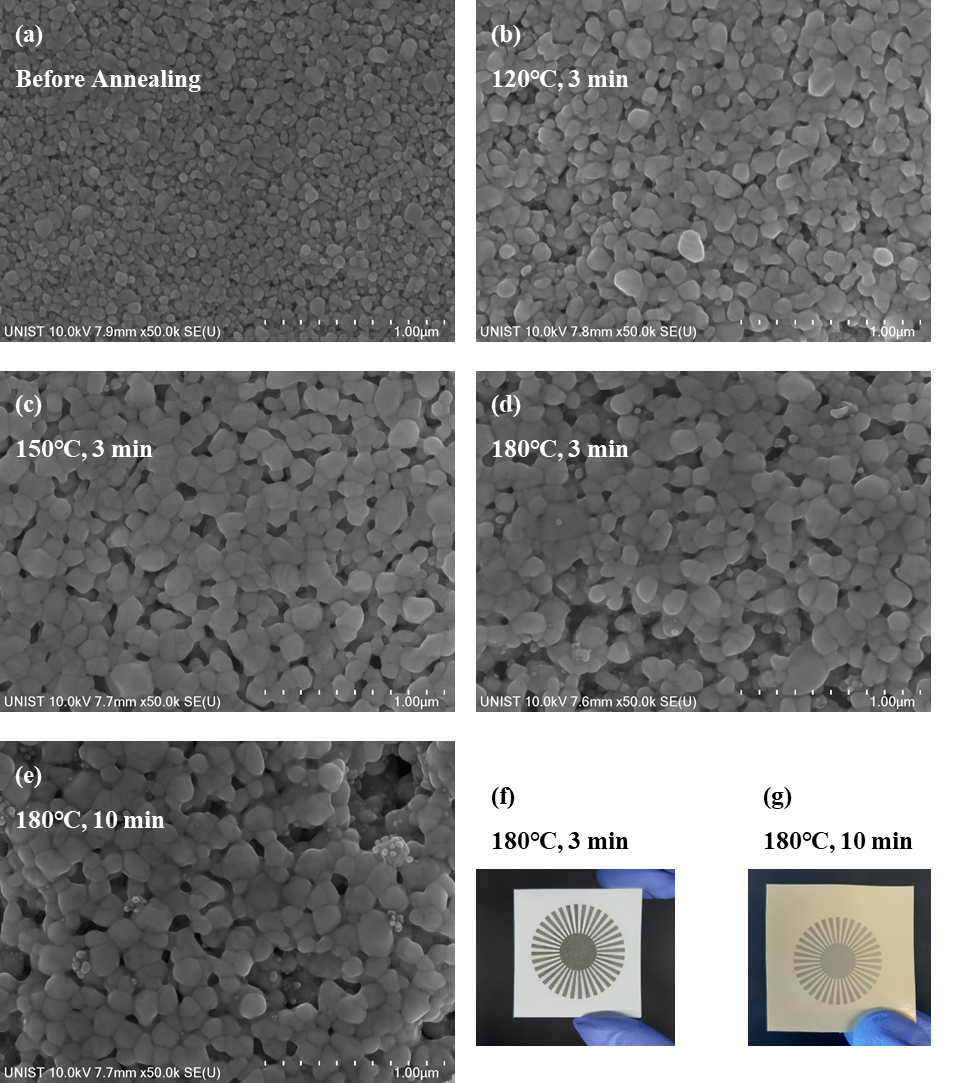


Figure S1. Scanning electron microscope (SEM) images of printed patterns under different annealing conditions. (a) Before annealing process. (b) At 120 ℃ for 3 minutes. (c) At 150 ℃ for 3 minutes. (d) At 180℃ for 3 minutes. (e) At 180 ℃ for 10 minutes. All the scale bars denote 1μm. (f) is a picture of the sample for the annealing condition (d). (g) is for the annealing condition (e).


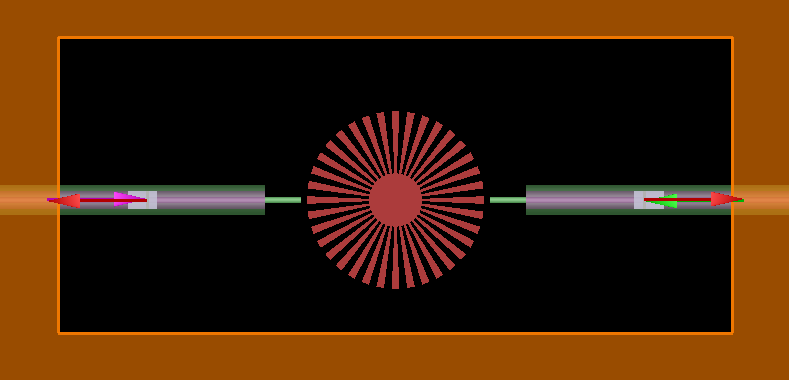


**PML (perfectly Matched Layer)**


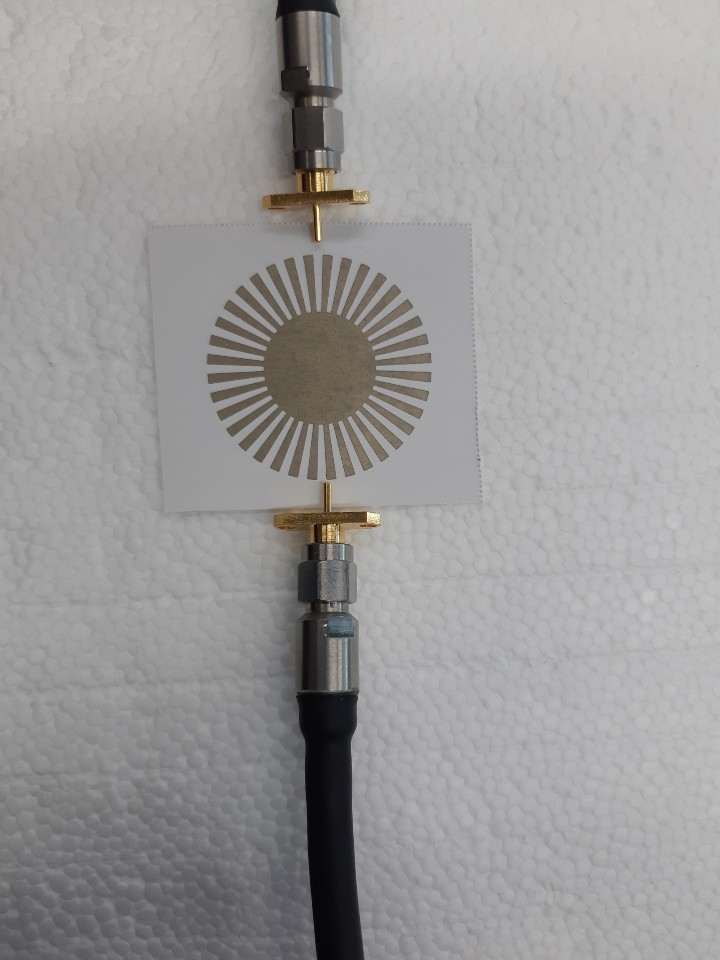


**VNA (Vector Network Analysis)**

**SMA connector**

**S1(source)**

**S2(detector)**


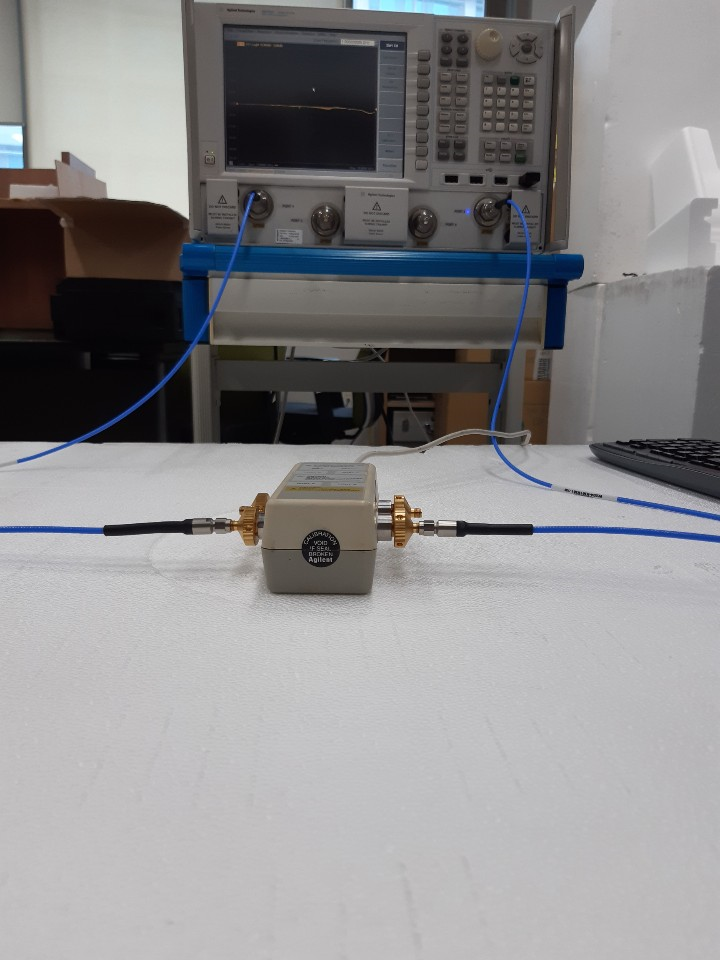


**Coaxial cable**

(a)

(b)

**Figure S2.** (**a**) Illustration of transmission simulations. Except for bending simulations, all simulated transmission spectra were obtained using finite difference time domain simulations (Lumerical FDTD). Source and detector probes (PortGroup) are located on the opposite side of the resonator. All four sides are surrounded by a perfectly matched layer (PML). The substrate was intentionally omitted in simulations to simplify the mesh condition. (**b**)

Schematic of the experimental setup. A coaxial probe, which consists of a coaxial cable and a SMA connector, was used for transmission measurements. The one end of the coaxial cable (Withwave, W204 Cable Assembly: Phase Stable Cable (DC to 40 GHz): 2.92 mm(Male) type) was connected to a VNA (Keysight Technologies, N5242A PNA-X), while the other was connected to the SMA connector (Pasternack W204 Cable Assembly: Phase Stable Cable (DC to 40 GHz): 2.92 mm(Male) type) for impedance matching. Two probes were deployed at opposite sides of the sample to measure the transmission amplitude (|S_21_|).

**
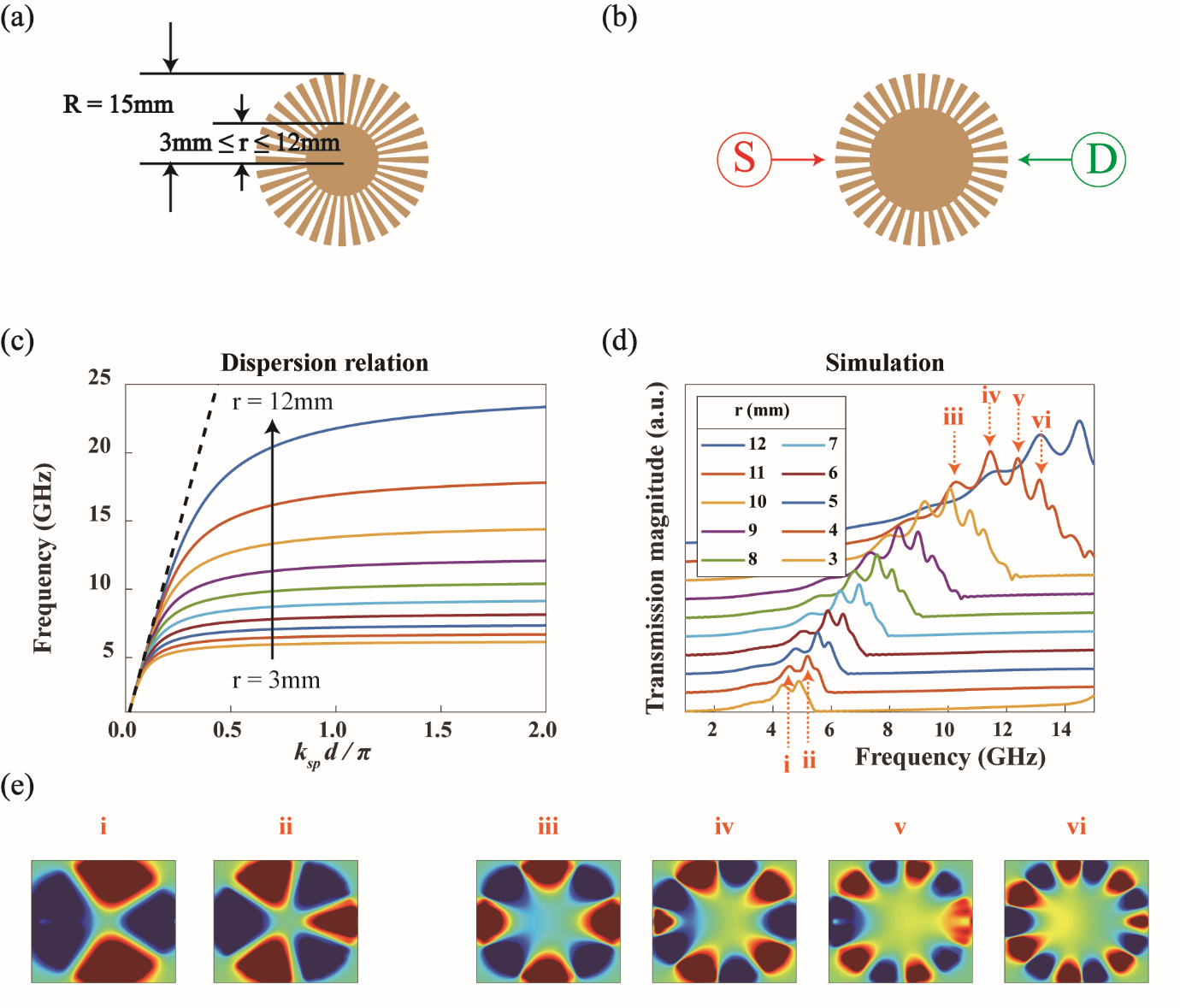
**

**Figure S3. (a)** and **(b)** Design and probe configuration for spoof plasmon resonators. **(c)** Dispersion relation from an analytic equation for an infinitely long (i.e., 2-dimensional) structure. The outer radius (*R*) is fixed as 15mm, while the inner radius (*r*) is varied from 3 mm to 12 mm by Δ*r* = 1 mm. The dashed line is a light line in air. **(d)** Simulated transmission spectra for ultrathin (i.e., 3-dimensional) structures. The spectrum is intentionally shifted upward. The arrows indicate the resonance peaks for *r* = 4 mm and 11 mm. **(e)** Field profiles at resonances indicated in **(d)** by arrows.


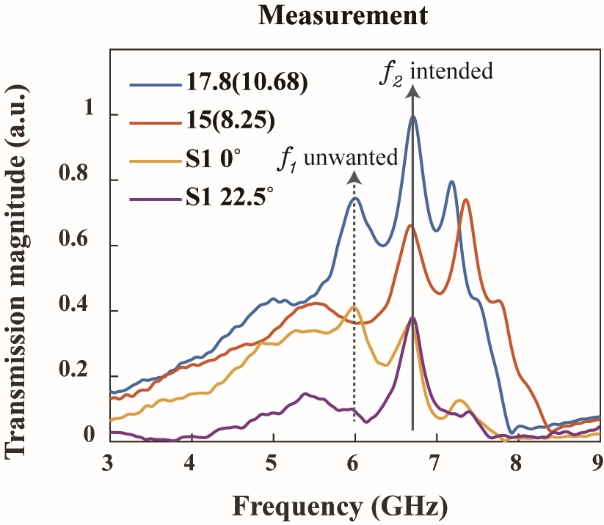


**Figure S4.** Vernier effect in a coupled plasmonic resonator which consists of larger (*R* = 17.8 mm, *r* = 10.68 mm) and smaller (*R* = 15 mm*,* *r* = 8.25 mm) resonators. Its design parameter corresponds to Figure 5 in main text. A slight mismatch between resonances in two single resonators is initially introduced to induce the amplitude tuning of the filtered peak, compared to the Figure 4 in the main text.

**
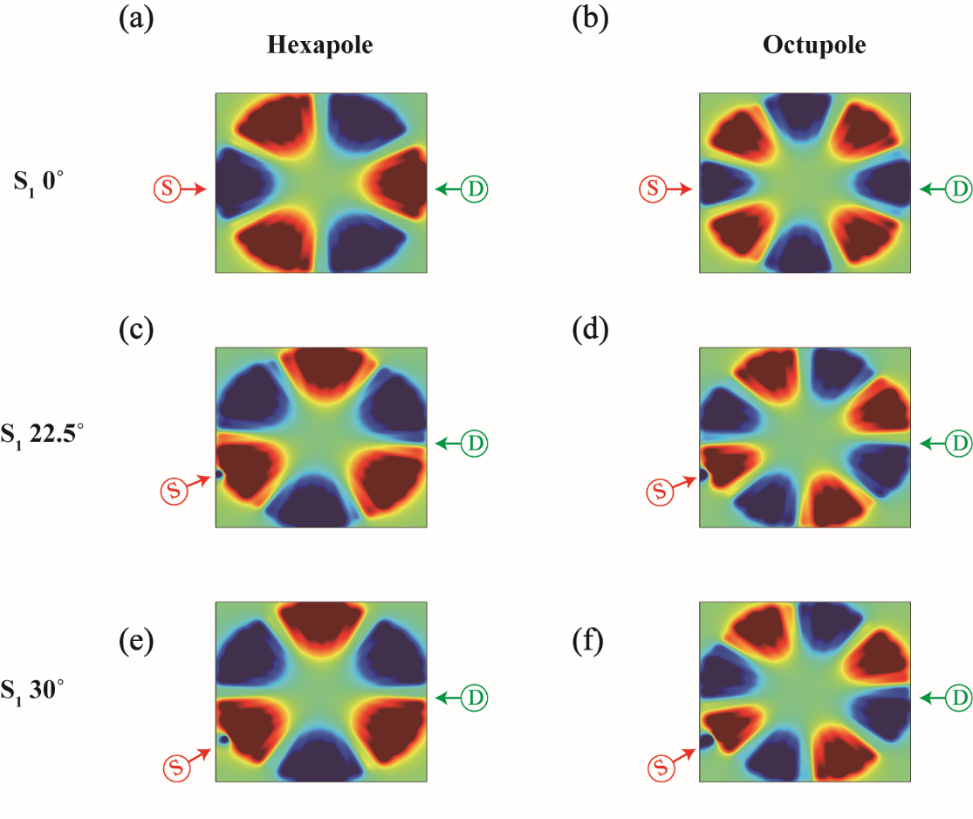
**

**Figure S5.** Field profiles of hexapole and octupole resonances for different source probe locations (*R* = 15 mm, *r* = 7 mm, and *N* = 36). Because the source probe drives the fields, the source probe location always becomes an antinode position (i.e., field maximum position). Therefore, when the source probe location is altered (from 0˚ to 22.5˚ and 30˚), the overall field profiles also rotate accordingly. **(a, b)** correspond to probes facing each other (S_1_: 0˚), (**c**, **d**) and (**e**, **f**) correspond to the source probe location of 22.5˚ and 30˚, respectively.

**Table S1.** Comparison of microwave resonators

| Reference | Resonator type | Substrate | Center f | Q-factor |
| --- | --- | --- | --- | --- |
| Our work | Spoof LSP | Photopaper (metal ink) | 6.58 GHz (octupole, experiment) | 31 (octupole, experiment) |
| [R1] | MIM ring LSP | Rogers RO4350 | 3.48 GHz (hexapole, simulation) | 69.6 (hexapole, simulation) |
| [R2] | Spoof LSP | RT 5880 | 3.43GHz (dipole, experiment) | 60 (dipole, experiment) |
| [R3] | MIM ring LSP | Rogers RO4350 | 11.04GHz (octupole, simulation) | 54.3 (octupole, simulation) |
| [R4] | Split ring resonator | Photopaper (metal ink) | 4 GHz | 14.3 (experiment) |

[R1] Yang, B. J., Zhou, Y. J. & Xiao, Q. X. Spoof localized surface plasmons in corrugated ring structures excited by microstrip line. *Opt. Express* **2015,** *23*, 21434-21442.

[R2] Gao, F., Gao, Z., Shi, X., Yang, Z., Lin, X. & Zhang, B. Dispersion-tunable designer-plasmonic resonator with enhanced high-order resonances. *Opt. Express* **2015,** *23*, 6896-6902.

[R3] Zhou, Y. J., Xiao, Q. X. & Yang, B. J. Spoof localized surface plasmons on ultrathin textured MIM ring resonator with enhanced resonances. *Sci. Rep.* **2015,** *5*, 14819.

[R4] Salim, A., Naqvi, A. H., Park, E., Pham, A. D., Lim, S., Inkjet printed kirigami inspired split ring resonator for disposable, low cost strain sensor applications. *Smart Mater. Struct.* **2020**, 29, 015016

Table S1 compares the existing microwave devices with our metal-ink printed plasmonic resonator. Because the silver inks we used have smaller conductivity than the more conventional copper lines on PCBs, the Q factor of our device is smaller than others. But, the Q factor and sensitivity of our device are still large enough to enable reasonably good sensing and spectral filtering.

In fact, our metal-ink printing of plasmonic resonators has *several unique merits* compared to other microwave resonator devices. (1) Our plasmonic resonators are printed using a common inkjet printer. Therefore, metal-ink printing can enable easy, cheap, and fast fabrication of various microwave components with adjustable design. (2) Our plasmonic resonators can be printed on a flexible, disposable substrate (including paper). This can reduce the production cost for sensing devices. (3) Our printed plasmonic resonators clearly show higher-order resonance modes that exhibit sharper spectral lines. This sharp spectral lines are desirable for many sensing and filtering applications.
